# Investigating the impact of couple counseling based on the CHARMS model on sexual quality of life and marital satisfaction of wives of men suffering from myocardial infarction: a study protocol

**DOI:** 10.1186/s12978-024-01776-3

**Published:** 2024-04-19

**Authors:** Soheila Rabeipoor, Kamal Khademvatani, Samira Barjasteh, Delniya Ghafuri

**Affiliations:** 1 Reproductive health, Reproductive Health Research Center, Clinical Research Institute, Urmia University of Medical Sciences, Urmia, Iran; 2 Department of Cardiology, School of Medicine, Urmia University of medical sciences, Urmia, Iran; 3 Reproductive Health Researcher Center, Clinical Research Center, Urmia University of Medical Sciences, Urmia, Iran; 4 Nursing & Midwifery Faculty, Urmia University of Medical Sciences, Campus Nazlu, 11KM Road Sero, Urmia, West Azarbaijan Iran

**Keywords:** Sexual counseling, Marital satisfaction, Sexual quality of life, Myocardial infarction

## Abstract

**Introduction:**

Cardiovascular diseases are a common chronic illness in adults, with implications for health and psychological well-being. These implications not only affect the patients themselves but also impact family members, especially the spouses of patients. One significant issue and consequence of this disease is its impact on marital relationships and sexual satisfaction, which can also influence other dimensions of quality of life. The aim of the current study is to determine the effect of couple counseling based on the CHARMS model on sexual quality of life and marital satisfaction of wives of men suffering from myocardial infarction.

**Method:**

This study is a clinical randomized controlled trial. Sampling will be done on a convenience basis. Participants will be randomly allocated into two groups: control (50 couples) and intervention (50 couples). Couples in 6 groups of 8 members each will attend counseling sessions based con the CHARMS model, with sessions held weekly and lasting for 60 min. Data collection tools will include Demographic information questionnaire, Women’s Sexual Quality of Life Questionnaire, Enrich Marital Satisfaction Questionnaire, Sexual Compatibility Questionnaire and Perceived Quality of Relationship Dimensions Questionnaire, which will be completed by women in both groups before and after the intervention. Data will be analyzed using appropriate statistical tests and SPSS software.

**Discussion:**

This trial will evaluate whether a counseling intervention based on the CHARMS model can enhance sexual quality of life and marital satisfaction of wives of men with myocardial infarction in Urmia city.

**Trial registration:**

IRCT code: IRCT20240218061046N1

## Abstract (plain language summary)

Cardiovascular diseases are a common chronic illness in adults, with implications for health and psychological well-being. One significant issue and consequence of this disease is its impact on marital relationships and sexual satisfaction, which can also influence other dimensions of quality of life.

This trial will evaluate whether a counseling intervention based on the CHARMS model can enhance sexual quality of life and marital satisfaction of wives of men with myocardial infarction in Urmia city. A CHARMS-based intervention with 4 principles addresses the sexual and marital relationship empowerment of couples following a severe heart attack. These principles include: (1) Counseling and providing information on the impact of cardiovascular diseases on sexual desires. (2) Counseling and providing information on a healthy sexual life and communication skills strategies with the sexual partner. (3) Counseling on uncovering false beliefs and misconceptions regarding relationship risks and fears. (4) Providing tips and solutions for resuming sexual relations after a severe heart event, addressing sexual and interpersonal challenges. This intervention sets patients’ expectations of sexual relationships based on a final focus on “sexual intimacy” as the ultimate goal of therapy.

This study is a clinical randomized controlled trial. Participants will be randomly allocated into two groups: control (50 couples) and intervention (50 couples). Couples in 6 groups of 8 members each will attend counseling sessions based con the CHARMS model, with sessions held weekly and lasting for 60 min.

## Introduction

Cardiovascular diseases are among the most prevalent chronic conditions and a leading cause of death and disability in adults globally [1]. The incidence of this disease doubles with each decade of life. Cardiovascular diseases, with an annual mortality rate exceeding 17 million people worldwide, rank first globally, particularly impacting low and middle income countries. It is projected that cardiovascular diseases will account for over 23 million deaths annually worldwide by 2030 [2]. Official data indicates a rising trend in mortality rates due to this issue in Iran as well [3]. Given the severe and chronic nature of cardiovascular diseases, they present a crisis in the affected individual’s life, significantly affecting not only their physical health but also their psychological well-being [4]. These diseases are recognized as key factors influencing individuals’ sexual lives in terms of quality, performance, and satisfaction. Sexual dysfunction and decreased sexual activity are prevalent among heart patients, impacting their overall sexual quality of life [5]. A primary sexual issue reported by heart patients is a decline or loss of sexual desire, often linked to concerns such as anxiety about sudden death during sexual activity, misinterpretation of normal signs of sexual arousal as cardiac symptoms like increased heart rate and breathing, and depression following a heart attack. Ultimately, this results in sexual dissatisfaction, poses risks to mental health, and consequently disrupts family unity [6].

The quality of marital relationships is a multidimensional concept that includes compatibility, sexual satisfaction, happiness, harmony, and commitment among couples. However, individuals with ischemic heart diseases may face challenges in their marital relationships due to fear and confusion about the impact of the illness. This can result in decreased satisfaction, reduced intimacy, and avoidance of social activities. Research indicates that the frequency of sexual relationships among patients with ischemic heart diseases can decrease by 40 to 70% [7].

Studies have indicated that ischemic heart diseases have a significant impact on the intimate relationships between couples. Many spouses of heart patients feel they are in a critical situation and want to reassure their partners of their love, but they are concerned about the consequences of sexual activity. This concern can result in the avoidance of sexual relationships, despite the importance of sexual activity to individuals’ quality of life [5, 7].

Patients with heart disease may be uncertain about the effects of sexual activity on the heart, and partners may generally avoid sexual intercourse due to fear of its consequences [8]. A decrease in the quality and frequency of sexual relationships has been observed in men following myocardial infarction (MI), along with a notable reduction in the frequency of sexual activity and sexual satisfaction [9]. Sexual dysfunction in men who have survived heart attacks can manifest as decreased libido, erectile dysfunction, and premature ejaculation [7]. Sexual education is crucial for patients and should commence in the acute phase of the disease, continuing throughout the recovery period. Studies have shown that men prioritize receiving information about the timing of sexual activity as part of their educational needs [10]. However, conversations about sexual matters among hospitalized cardiac patients are often stifled by shame, modesty, and cultural barriers, resulting in the neglect of sexual counseling, which is a vital aspect of nursing counseling [11].

Since all stages of sexual activity are accompanied by an increase in heart rate, which can lead to the manifestation of a heart attack experience and fear of a recurrent heart attack in patients, preventing such challenges and sexual concerns in patients requires creating awareness and counseling to explore and address sexual anxieties [12]. However, sexual rehabilitation after heart disease has been largely overlooked, and even discussing sexual issues during medical visits is perceived as difficult by both patients and physicians. Studies indicate that recommendations given to patients regarding resuming sexual activity after acute cardiac events or surgery are variable and insufficient, leading many patients to struggle with the fear of engaging in sexual intercourse [13] Therefore, it is essential to provide appropriate planning and sexual counseling tailored to each patient’s condition to enhance the sexual health of cardiac patients. The use of sexual counseling methods helps patients maintain their emotional relationship with their spouse despite all the physical and psychological changes related to their conditions and enables them to have a satisfying sexual life [14].

Models are guidelines that assist healthcare providers in implementing appropriate and effective supportive interventions and solutions for health concerns and issues such as sexual problems in patients [15]. One of the activities that should be included in cardiac rehabilitation programs is sexual counseling within the framework of cardiac rehabilitation programs. The goal of providing sexual counseling to cardiac patients is to assess existing sexual problems, provide information about concerns and safety in sexual activity after a cardiac event or process [16]. The American Heart Association and the European Society of Cardiology recommend that all patients with cardiovascular diseases should be evaluated for sexual concerns and offered free sexual counseling. Among the recommended counseling methods by these associations that can be helpful is the CHARMS-based counseling method (Cardiac Health and Relationship Management and Sexuality) [17].

A CHARMS-based intervention with 4 principles addresses the sexual and marital relationship empowerment of couples following a severe heart attack. These principles include: (1) Counseling and providing information on the impact of cardiovascular diseases on sexual desires. (2) Counseling and providing information on a healthy sexual life and communication skills strategies with the sexual partner. (3) Counseling on uncovering false beliefs and misconceptions regarding relationship risks and fears. (4) Providing tips and solutions for resuming sexual relations after a severe heart event, addressing sexual and interpersonal challenges. This intervention sets patients’ expectations of sexual relationships based on a final focus on “sexual intimacy” as the ultimate goal of therapy [18].

Considering the prevailing culture in Iranian families where marital relationships are perceived as personal and private, the quality of sexual relationships is not usually assessed in medical treatment programs or even in the follow-ups of cardiac patients. Studies conducted in Iran descriptively examine the sexual problems of cardiac patients, with few intervention studies focusing on the impact of treatment methods on their sexual quality of life.

### General objective

To determine the impact of couple counseling based on the CHARMS model on sexual quality of life and marital satisfaction of wives of men with myocardial infarction.

### Specific objectives

- To determine and compare the average score of sexual quality of life of wives of men with myocardial infarction before and after the intervention in two groups.
- To determine and compare the average score of marital satisfaction of wives of men with myocardial infarction before and after the intervention in two groups.

### Practical objective

It is expected that through the current research and confirmation of the effects of couple counseling based on the CHARMS model on sexual quality of life and marital satisfaction of wives of men with myocardial infarction, this method and intervention will be utilized by women’s health and midwifery specialists, maternity experts, and nurses working in healthcare centers. This will enable an important step towards improving the sexual quality of life and marital satisfaction of these wives through the use of models and guidelines.

### Research hypothesis

Couple counseling based on the CHARMS model leads to an increase in the average score of sexual quality of life and marital satisfaction in wives of men with myocardial infarction.

## Methods

The present study is a clinical trial aimed at determining the impact of couple counseling based on the CHARMS model on sexual quality of life and marital satisfaction of wives of men with myocardial infarction in Urmia city.

### Inclusion and exclusion criteria

The inclusion criteria for couples in the study include: definite diagnosis of heart attack and a minimum of 6 months since its occurrence in men, first-time heart attack in men, living with the spouse during the study period, no history of mental or emotional trauma in the past 6 months, no history of chronic disease in women, having children, residing in Urmia city, having at least primary education, age between 35 and 55 years in spouse, and not participating in similar research projects in the past three months. The exclusion criteria include not attending counseling sessions more than once and withdrawing from continued cooperation and changing the city of residence during the study period.

### Sample size

According to the study by Baqeri et al. [11], the average sexual satisfaction of spouses in the intervention group before intervention was 2.47 ± 81.30 and after intervention was 2.28 ± 82.70. Considering an alpha of 0.05 and 80% power, the sample size was estimated to be 45 couples in each of the two intervention and control groups. Taking into account potential sample attrition in each study arm (intervention and control), approximately 50 couples will be selected and included in the study.

$$=\frac{{\left({z}_{1-\frac{\propto }{2}}+{z}_{1-\beta }\right)}^{2}\times {{\sigma }_{1}}^{2}+{{\sigma }_{2}}^{2}}{{\left({\mu }_{1}-{\mu }_{2}\right)}^{2}}=\frac{{\left(1.96+0.58\right)}^{2}\times {2.28}^{2}+{2.47}^{2}}{{(82.70-81.30)}^{2}}=\frac{7.89\times 5.19+6.10}{1.96}=45$$

### Data collection tools

The following questionnaires will be used to collect data in this research.

Demographic information questionnaire: This tool includes demographic characteristics of the participants such as age, spouse’s age, duration of marriage, number of children, education level, spouse’s education level, employment status of the couple, family income sufficiency, current contraceptive method, duration of illness, age of first heart attack, medications used, and stage of heart disease in men.

Women’s Sexual Quality of Life Questionnaire: This tool measures the relationship between sexual dysfunction and women’s quality of life. It consists of 18 items divided into four main sections including psychological-sexual feelings, satisfaction with relationship and sexual activity, feelings of worthlessness, and sexual suppression. Scoring is based on a Likert scale ranging from 1 to 6, indicating from strongly agree to strongly disagree. The total score will range from 18 to 108, where a higher score reflects higher sexual quality [19, 20].

Enrich Marital Satisfaction Questionnaire, consisting of 47 questions. The minimum score is 47 and the maximum is 235. Scores below 115 indicate low marital satisfaction, 116 to 230 indicate moderate satisfaction, 231 to 345 indicate high satisfaction, and 346 to 460 indicate ideal satisfaction [21].

Sexual Compatibility Questionnaire: This questionnaire consists of 5 questions and aims to assess the level of sexual compatibility between couples. It is scored on a 5-point Likert scale. Higher scores indicate higher sexual compatibility. Scores below 8 indicate low sexual compatibility, scores between 9 and 16 indicate moderate compatibility, and scores above 17 indicate high sexual compatibility [22].

Perceived Quality of Relationship Dimensions Questionnaire: This scale consists of 18 questions and 6 subscales evaluating satisfaction, commitment, intimacy, trust, passion, and love. The minimum score is 18 and the maximum is 126. Lower scores indicate lower quality and higher scores indicate better quality of marital relationship in different dimensions [23, 24].

### Validity and reliability of questionnaires

Women’s Sexual Quality of Life Questionnaire: This tool has been translated, validated, and culturally adapted in Iran by Masoumi et al. (2013), demonstrating good validity, high internal consistency, and high reliability. It had a Cronbach’s alpha coefficient of 0.73 and an internal consistency of 0.88 (ICC = 0.88) [25].

The Enrich Marital Satisfaction Questionnaire: The validity and reliability of this tool in Iran were confirmed by Masoumi et al. (2021) with a validity of 0.86 and a reliability of 0.80 [21].

The Sexual Compatibility Questionnaire: In the study by Rashan Chesli et al. (2014), formal and content validity were assessed and approved by experts and professors. The reliability was calculated using Cronbach’s alpha, yielding a value of 0.91 [22].

The Perceived Quality of Relationship Dimensions Questionnaire: This scale, translated by Nilforoushan et al., had its content validity confirmed with a Cronbach’s alpha of 0.95 [24].

### Sampling and data collection

Samples will be selected using convenience sampling and divided into two groups: intervention (45 couples) and control (45 couples) based on even and odd numbers in sealed envelopes. The participants will be asked to choose an envelope, in such a way that even numbers inside the envelope correspond to the intervention group and odd numbers belong to the control group.

Researchers will visit the Seyyed Al-Shohada Hospital in Urmia after obtaining official approval from the Ethics Committee of Urmia University of Medical Sciences. After introducing the research, explaining the objectives and methods to the patients, eligible individuals will be selected. Upon their agreement to participate, informed written consent will be obtained from both men and their spouses. Subsequently, the couples’ phone numbers will be collected for scheduling counseling sessions. Researchers will emphasize the importance of couples attending counseling sessions together.

A couple participated in sessions within 6 groups, each consisting of 8 members, held weekly for 60 min. After collecting participants’ phone numbers, it was arranged for them to be prepared for 6 sessions of sexual counseling based on the CHARMS method. The intervention program was designed based on the CHARMS empowerment program and following similar studies [17].This intervention includes: (1) sexual education and counseling for spouses of patients with heart attacks, (2) educational and supportive intervention for heart attack patients by researchers and nurses in the cardiac rehabilitation program, (3) delivering informative booklets to patients, and (4) providing pamphlets on the topics of each session to increase awareness among heart attack patients and their spouses.

The content of each counseling and educational session was compiled by the research team from books, articles, websites, and reputable sources such as: European Society of Cardiology and American Heart Association [26–31] (Table 1).

After preparing the counseling content for each session, we validated the developed content using the Delphi method based on feedback from reproductive health and cardiology experts at Urmia University of Medical Sciences. These experts possessed the necessary knowledge and skills in sexual counseling and essential care for heart patients. We conducted three rounds of questionnaires with a panel consisting of three cardiology specialists and three reproductive health specialists.

Interventions at the Cardiac Rehabilitation Center of Seyed Al-Shohada Hospital will be conducted with the presence of patients suffering from heart attacks and their spouses. The intervention will be in the form of discussions, group consultations, and individual consultations if needed, which will be presented at the end of the session.

The sessions will be managed by researchers who have the necessary skills and knowledge regarding sexual counseling. The topics of each session will be presented to the participants by the researchers. In this regard, the researcher will facilitate and guide the sessions. During the intervention, tools such as pamphlets, PowerPoint presentations, speeches, and couples counseling will be used to enhance the effectiveness of the intervention program.

First, the demographic information form is completed by the control and intervention groups, and then other questionnaires are completed by the women of both groups (pre-test). One month after the end of the intervention, the questionnaires will be completed again by women in the control and intervention groups (post-test).

**Table 1:** Contents of counseling sessions based on the CHARMS method

No	The content of the session	Type of consultation
First	With a focus on establishing communication and building trust and collaboration between couples, evaluating the mental, emotional, and physical health of individuals, identifying their sexual problems, informing couples about the session structure and setting goals, educating on the sexual cycle, and familiarizing them with sexual performance and the internal and external organs of men and women. Defining sexual desire, arousal, lubrication, and orgasm, as well as familiarizing them with the sexual response pattern and explaining the physiological changes in men and women during sexual activity, including sexual positions.	Group
Second	Counseling and providing information about cardiovascular diseases and symptoms, general and sexual consequences on the individual and their impact on the life of patients.	Group
Third	Counseling and providing information on the impact of cardiovascular disease on reducing sexual desire, lack of sufficient communication skills in sexual relationships, misconceptions about sexual relationships, providing information and education on coping with shame and deciding to resolve it, providing information on sexual aspects of life and training strategies to enhance marital satisfaction, providing necessary information and education on sensory focus techniques. Providing information on expressing feelings and beliefs about sexual behaviors and teaching problems-solving skills.	Group
Fourth	Consultation and provision of information on identifying the root causes of decreased sexual desire, offering solutions, strengthening empathetic communication, sexual discourse with a partner, explaining the secrets of sexual attraction, assisting in maintaining verbal and emotional relationship with a spouse, emphasizing the importance of lovemaking and romance, warning signs of heart disease during or after intimacy.	Group
The fifth	Collaborative contract writing technique to articulate needs and expectations in sexual relationships, clarifying expectations, correcting misconceptions and myths about sexual relationships, guidance on creating a structured timeline for noting thoughts and mindset during desirable and unpleasant sexual experiences, aversion, muscle tension, and emotional pressures. Household responsibilities, addressing potential inquiries.	Group
The sixth	Review of previous materials and nutritional recommendations, sleep hygiene education, performing stretching exercises and sports, recommending daily walks, bedroom environment, teaching alternative positive thinking and stopping negative thoughts.	Group

### Data analysis

Descriptive analysis will be utilized to present baseline characteristics. Independent t-tests/Mann-Whitney tests will be employed to compare normal/abnormal quantitative variables between two groups, while paired t-tests/Wilcoxon tests will assess the intervention’s impact on normal/abnormal quantitative variables. Chi-square tests will be used to compare qualitative variables between groups, and Mac-Nemar tests will evaluate the intervention’s effect on qualitative variables. The Kolmogorov-Smirnov test will assess the normality of data distribution for quantitative variables. If needed, other statistical tests like regression models may be applied. Statistical analyses will be conducted using SPSS 22, with a significance level of 5% considered.

## Discussion

One challenge faced by post-heart attack patients is sexual activity. Lack of awareness about this issue can lead to problems like rehospitalization, sudden death during sexual activity, and sexual dissatisfaction for both the patient and their partner [5, 6]. After overcoming the heart attack crisis and its treatment, couples aim to address the sexual gaps in their lives. However, they may encounter sexual difficulties, emotional upheavals, and changes in intimacy levels post-recovery. Men may struggle with hidden fears, while women fear that requests for sexual intimacy may be seen as carelessness or misplaced expectations post-illness. This dysfunctional cycle can strain families and couples for years, impacting their sexual quality and marital satisfaction. Therefore, providing sexual education and counseling can enhance their sexual quality of life.

After this study, an improvement in the quality of sexual life and marital satisfaction of men with myocardial infarction can demonstrate the benefits of sexual counseling intervention based on the CHARMS model in enhancing their sexual quality of life. Similarly, negative results can help in designing new strategies. Additionally, these findings can contribute to the content used in future programs and studies.

## Data Availability

Not applicable
